# Assessment of Glycemic Control at St. Luke’s Free Medical Clinic: Retrospective Chart Review

**DOI:** 10.2196/31123

**Published:** 2022-01-24

**Authors:** Wade Hopper, Justin Fox, JuliSu Dimucci-Ward

**Affiliations:** 1 Department of Surgery Edward Via College of Osteopathic Medicine - Carolinas Spartanburg, SC United States; 2 Department of Preventive Medicine and Public Health Edward Via College of Osteopathic Medicine - Carolinas Spartanburg, SC United States

**Keywords:** free clinic, glycemic control, underserved, uninsured, diabetes, health care delivery, treatment program

## Abstract

**Background:**

A free clinic is a health care delivery model that provides primary care and pharmaceutical services exclusively to uninsured patients. With a multidisciplinary volunteer clinical staff, which includes physicians, social workers, dieticians, and osteopathic medical students, St. Luke’s Free Medical Clinic (SLFMC) cares for over 1700 patients annually in Spartanburg, South Carolina.

**Objective:**

This study aims to measure the change, over time, in patient hemoglobin A_1c_ measurements at the SLFMC to quantify the success of the clinic’s diabetes treatment program.

**Methods:**

A prospective-retrospective chart review of patients (n=140) enrolled at the SLFMC between January 1, 2018, and January 1, 2021, was performed. Patients were stratified as having controlled (hemoglobin A_1c_<7.0, n=53) or uncontrolled (hemoglobin A_1c_≥7.0, n=87) diabetes relative to a therapeutic hemoglobin A_1c_ target of 7.0, which is recommended by the American Diabetes Association. For both controlled and uncontrolled groups, baseline hemoglobin A_1c_ values were compared to subsequent readings using a Wilcoxon matched-pairs signed rank test. Results from the SLFMC population were compared to the published literature on hemoglobin A_1c_ from other free clinics.

**Results:**

Patients with uncontrolled diabetes experienced significant reductions in median hemoglobin A_1c_ at both 6 months (*P*=.006) and 1 year (*P*=.002) from baseline. Patients with controlled diabetes showed no significant changes. Black and Hispanic patients with uncontrolled diabetes experienced a 1.0% mean improvement in hemoglobin A_1c_ over the study window. The SLFMC’s wholly uninsured patient population showed a population rate of controlled diabetes (42%), which was similar to recent nationwide averages for adults with diabetes (51% to 56%), as reported by the National Health and Nutrition Examination Survey. The clinic’s Hispanic population (n=47) showed the greatest average improvement in hemoglobin A_1c_ of any ethnic group from baseline. Additionally, 61% of the SLFMC’s Black population (n=33) achieved a hemoglobin A_1c_ of <7.0 by the end of the study window, which surpassed the nationwide averages for glycemic control.

**Conclusions:**

We present free clinic hemoglobin A_1c_ outcomes obtained through a retrospective chart review. Uninsured patients treated for diabetes at the SLFMC show a reduction in hemoglobin A_1c_, which is comparable to nationwide standards, although average hemoglobin A_1c_ levels in this study were higher than nationwide averages. Black and Hispanic patients with uncontrolled diabetes showed a mean 1% improvement in hemoglobin A_1c_ levels. These results represent some of the first in the literature emerging from a free clinic that is not affiliated with a major medical school.

## Introduction

Free medical clinics are private nonprofit practices that provide a health care safety net for uninsured individuals in a community. Free clinics provide essential primary care services that include minimal paid staff and maximize the use of a volunteer clinical staff [1]. According to the Institute of Medicine, safety net practices are defined as “those providers that organize and deliver a significant level of health care and other needed services to uninsured, Medicaid, and other vulnerable patients” [2-4]. Free clinics seem to have arisen in response to specific populations’ health care needs that are not satisfied by existing public or private programs [5]. Approximately 1400 free clinics provide care to over 2 million Americans each year [5]. Free clinics rely on charitable donations and grants to fund their daily practices, as most free clinics receive no federal funding [1,6]. In 2020, the National Association of Free Clinics (NAFC) formalized quality-of-care standards for all member associations [7]. Outcome reporting is becoming increasingly critical for individual free clinics to sustain growth through charitable donations.

An understanding of free clinic patients, practices, and outcomes can yield important insights into the efficacy of free clinics compared to that of other safety net institutions. Recent research by Julie Darnell has elucidated some commonalities across free clinics [1,5]. Namely, most of them are young: nearly 90% of existing free clinics were founded more recently than 2000 [5]. Furthermore, almost all free clinics rely upon volunteer health practitioners to provide the bulk of their care [1]. Many outcome studies have been published, which outlined free clinics’ management of chronic disease [8-13]. Most publications in the free clinic sector represent student-ran, school-affiliated clinics. As entities, most free clinics have little to no incentive to publish outcomes data—after all, they do not receive government funding or participate in traditional insurance models. Moreover, reliance on volunteer staff places a human resources constraint on the publishing power of free clinics. This study represents one of the first of its kind to emerge from a free clinic that is not run by medical students.

The uninsured population is generally underrepresented in the medical literature. In 2019, over 26 million American adults—8.0% of the population—lacked health insurance [14]. An estimated 13%-16% of South Carolina adults aged 18-64 years lacked health insurance as of 2019 [15,16]. It is known that being uninsured places people at risk for incurring health care–associated debt, thereby delaying treatment [17]. Black and Hispanic people are overrepresented in the uninsured pool compared to the general population [18]. Despite the implementation of the Affordable Care Act, approximately 1 in 10 nonelderly Americans remained uninsured at the start of the 2020s [19]. Additionally, health concerns of the uninsured hold current relevance owing to the COVID-19–related economic shutdown in the United States. Health insurance in the United States is often tied to employment. Early during the COVID-19 pandemic, unemployment claims peaked in April 2020 at 36.5 million—14.7% of the population—and the largest figure observed since such records began [20,21]. Because free clinics exclusively serve the uninsured, research in this sector helps characterize the health behaviors and outcomes of uninsured Americans.

In 2020, St. Luke’s Free Medical Clinic (SLFMC) enrolled 1700 patients and averaged about 140 visits per week (personal communication from Patricia Whitney, SLFMC clinic director, May 2021). Of these 1700 patients, 920 (54%) were non-White. The clinic annually receives over 7000 hours of help from over 50 clinical volunteers, including physicians, mid-level practitioners, nurses, pharmacists, social workers, and a dietician. Patients generally see the same providers on a recurring basis, and this facilitates long-term clinical relationships. There are two clusters of food deserts in Spartanburg which span 8 of the city’s 20 census tracts [22]. The SLFMC provides a food bank and transportation assistance to patients with needs in those areas. The SLFMC’s licensed dispensing pharmacy is stocked with a wide variety of generic medications, and in 2020, upward of US $6 million worth of medications were provided by the clinic at no or minimal cost to patients. Further, of note, the SLFMC provides in-house language interpretation and translation services. Some of the clinic’s 334 Hispanic patients exclusively speak Spanish, and language assistance played a part in over 300 visits in 2020.

The SLFMC’s patients apply for and are awarded contracts of care based on financial need. Patients may continue receiving care so long as they lack health insurance—if a patient becomes old enough to qualify for Medicare, for example, or if a patient finds insurance through an employer, then the SLFMC will rotate that patient out of their care to free up space for awaiting applicants. Type 2 diabetes mellitus (T2DM) is the most prevalent diagnosis at the SLFMC. SLFMC patients diagnosed with T2DM often have a few other diagnoses for which they are receiving appropriate care. Patients usually see the same physician or nurse practitioner on a recurring basis. Psychiatrists, dieticians, and social workers are also involved in diabetes care teams. Patients with diabetes receive counseling in accordance with the Association of Diabetes Care and Education Specialists’ ADCES7 Self-Care Behaviors guidelines. Diabetes-specific pharmacotherapy was initiated as seen fit per practitioner. Finally, diabetes-specific follow-up visits typically occur every 3 to 6 months depending on the patient’s glycemic control.

This study attempts to answer a handful of questions: what is the quality of diabetes care at the SLFMC as measured by glycemic control over time? With respect to hemoglobin A_1c_, how does treatment at the SLFMC measurably compare to that provided by other free clinics? How do hemoglobin A_1c_ levels at the SLFMC compare to nationwide averages? Finally, do the clinic’s hemoglobin A_1c_ outcomes differ depending on race or Hispanic ethnicity?

## Methods

### Methods Overview

Study methods were approved by the institutional review board at Edward Via College of Osteopathic Medicine. A retrospective chart review was used to gather data. This method is well-established in free clinic literature as being a cost- and time-efficient means of reporting outcomes and comparing them to existing professional standards [8-13]. The use of hemoglobin A_1c_ as a diabetes quality-of-care metric is endorsed by the US government and the American Diabetes Association (ADA) [23-25]. Hemoglobin A_1c_ is an indicator of 3-month average blood glucose as well as a predictor of diabetes-related morbidity and microvascular complications [26-28]. The ADA endorses a hemoglobin A_1c_ threshold of 7.0 as being appropriate for most adults [23].

### Study Population

All patients’ medical charts at the SLFMC between January 1, 2018, and January 1, 2021, were screened for a diagnosis of T2DM. Inclusion criteria were as follows: (1) a diagnosis of diabetes, prediabetes, hyperglycemia, or metabolic syndrome; and (2) two or more different hemoglobin A_1c_ levels documented, with at least 6 months between the first and last recorded hemoglobin A_1c_ levels. Patients with a documented pregnancy within the study timeframe were excluded owing to the potential confounding effect of gestational diabetes. Patients with diabetes were stratified into two groups: controlled (baseline hemoglobin A_1c_<7.0) and uncontrolled (baseline hemoglobin A_1c_≥7.0) [12,13]. Stratification was essential because the two groups have two different therapeutic goals: the goal when treating controlled diabetes is to maintain hemoglobin A_1c_ levels under 7.0, while the goal when treating uncontrolled diabetes is to reduce hemoglobin A_1c_ levels to <7.0 [23].

### Data Collection

All available descriptive data and hemoglobin A_1c_ readings—including dates—for individual patients were transcribed from a hard copy medical record into Microsoft Excel. Descriptive data included age, gender, race/ethnicity, and BMI. Smoking status and the presence of concomitant diagnosis with essential hypertension (ICD-10-CM I10) were also recorded. Characteristics of the SLFMC sample were compared to data from Spartanburg County to explore what kinds of patients the SLFMC attracts [29,30]. Baseline hemoglobin A_1c_ values were defined as each patient’s first recorded hemoglobin A_1c_ value within the study window. To facilitate comparison, subsequent hemoglobin A_1c_ values of each patient were rounded to either 6 months, 1 year, or 2 years from baseline. The baseline hemoglobin A_1c_ value was also used to sort individuals into 1 of 2 groups on the basis of whether they had controlled (hemoglobin A_1c_<7.0) diabetes or uncontrolled (hemoglobin A_1c_≥7.0) diabetes relative to a best-practice hemoglobin A_1c_ treatment goal of 7.0, as recommended by the ADA [23]. Finally, each patient’s most recent hemoglobin A_1c_ values within the study timeframe were recorded. The most recent hemoglobin A_1c_ value on file was necessary for comparing treatment efficacy because not all patients received treatment for the same duration—some of the participants enrolled at the SLFMC in the middle of the study’s timeframe, and some patients also left the clinic outright or aged out into Medicare.

### Outcomes

The primary outcome measured per individual was the net change in hemoglobin A_1c_ (△A_1c_) from baseline to the most recent visit on record. At the group level, successful treatment was defined as maintenance of hemoglobin A_1c_ in patients with controlled diabetes and reduction in hemoglobin A_1c_ in those with uncontrolled diabetes. The primary outcome measured per group was median hemoglobin A_1c_. Finally, the success of the SLFMC diabetes treatment program was assessed by stratifying the clinic’s A_1c_ outcomes into three tiers of <7.0, 7.0-8.0, and >8.0. Proportions of patients within each range were compared to the hemoglobin A_1c_ data from various populations in the recent literature.

### Statistical Analysis

A Wilcoxon matched-pairs signed rank test was performed for the median group’s hemoglobin A_1c_ outcomes (controlled vs uncontrolled diabetes). The hemoglobin A_1c_ distribution was expected to show a skew, so the median value was chosen over the mean value to dampen the influence of outliers. A Bonferroni-Dunn post-test was applied to the resultant *P* values to standardize them to a single significance level (a) per group. Between-group medians, *P* values of <.05 were considered significant. Graphs were generated using GraphPad Prism (version 9; GraphPad Software, Inc).

## Results

**Figure 1:**
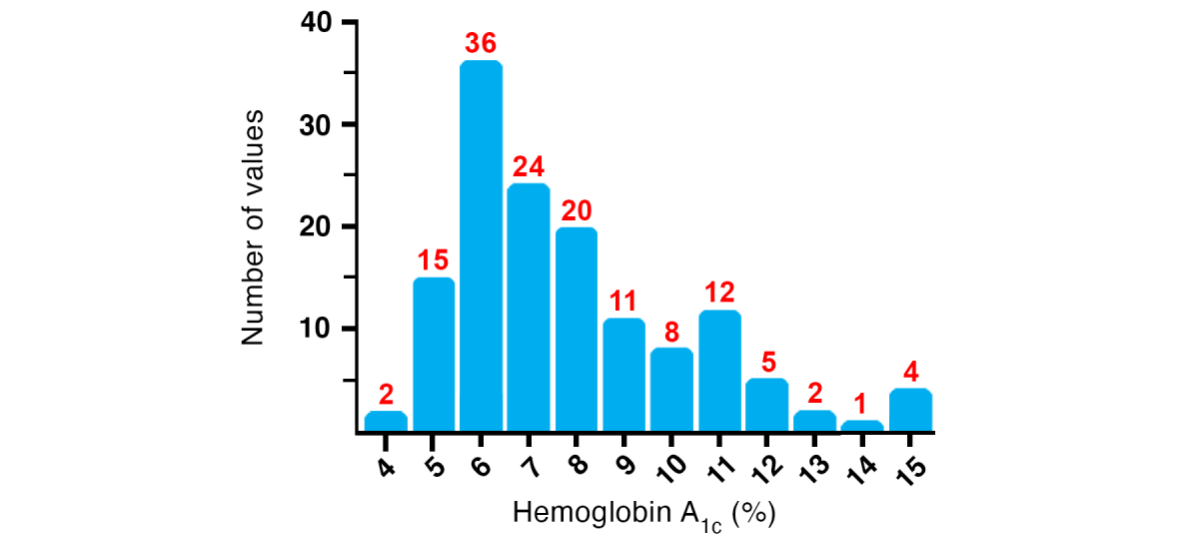
Baseline hemoglobin A_1c_ values for all 140 patients ranged from 4.4 to 15.8. Columns indicate ranges centered at the halfway point of each integer; column at 5, for example, contains all baseline hemoglobin A_1c_ values between 5 and 5.99. The number of patients within each hemoglobin A_1c_ range at baseline is indicated in red above each column.

This study examined 140 individuals who had the clinical diagnosis of T2DM and were treated at the SLFMC. Of them, 38% (n=53) of patients had controlled diabetes and 62% (n=87) had uncontrolled diabetes at baseline. A demographic profile (n=140) is detailed in Table 1. The study group had a high proportion of Hispanic patients (34%) and a low proportion of Caucasian patients (41%) compared to county data (7% and 73%, respectively). High rates of comorbid obesity (61%) and hypertension (74%) were also observed at rates of nearly twice the local prevalence for these diagnoses. The mean hemoglobin A_1c_ value at baseline was 8.3 (SD 2.5). The distribution of hemoglobin A_1c_ values at baseline is presented in Figure 1.

As a group, patients with controlled diabetes were defined by a baseline hemoglobin A_1c_ value of <7.0. According to the most recent observations, 75% of the patients with controlled diabetes remained controlled hemoglobin A_1c_ values and 25% experienced increases in hemoglobin A_1c_ values, which transitioned them to the uncontrolled category (Table 2). Conversely, patients with uncontrolled diabetes were defined by baseline hemoglobin A_1c_ values of ≥7.0. At 1 year from baseline, 25% of patients with uncontrolled diabetes experienced reductions in hemoglobin A_1c_ values, which transitioned them to the controlled category. This proportion declined over time to 21% according to most recent data.

**Figure 2:**
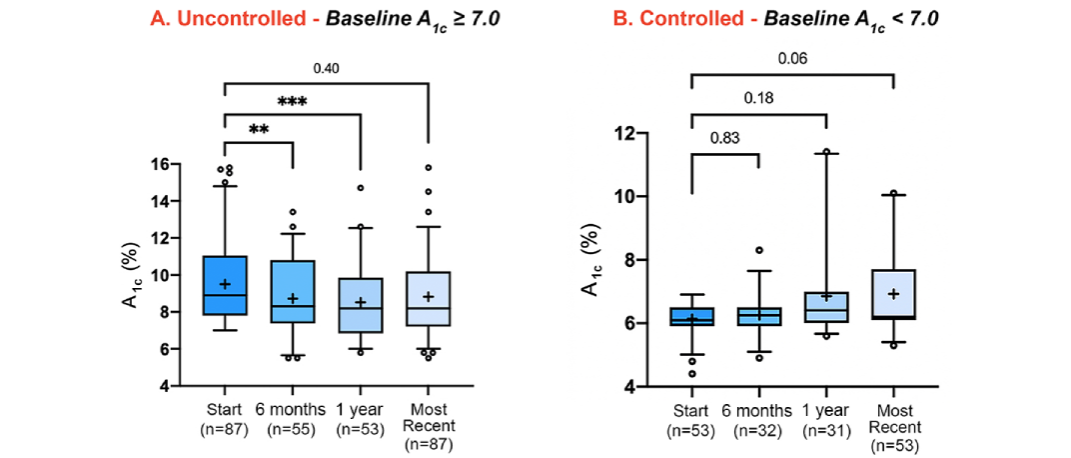
Boxes represent IQRs (25th-75th percentile) of hemoglobin A_1c_ values from patients with uncontrolled type 2 diabetes mellitus (A; left panel) and controlled type 2 diabetes mellitus (B; right panel) at baseline. Whiskers indicate 2 SDs from mean values (5%-95%). Lines within boxes represent median values. The “+” sign indicates mean values. Open dots represent outliers 2 SDs from the mean. **P=.006 ***P=.002.

Patients with uncontrolled diabetes experienced a significant reduction in median hemoglobin A_1c_ values at both 6 months (*P*=.006) and 1 year (*P*=.002) from baseline (Figure 2A). Within this group, both mean and median hemoglobin A_1c_ values decreased compared to baseline at every measurement interval. Patients with controlled diabetes showed no significant changes in median hemoglobin A_1c_ values (Figure 2B). Within this group, both mean and median hemoglobin A_1c_ values increased compared to baseline at every measurement interval. These data support the hypotheses that patients with uncontrolled diabetes would experience significant changes in median hemoglobin A_1c_ values, whereas those with controlled diabetes would not.

**Table 1:** Patient demographics.

Characteristics		St. Luke’s Free Medical Clinic (N=140)	Spartanburg County [24,25]
Age (years), mean (SD)		52.4 (10.2)	—^a^
**Sex, n (%)**			
	Male	62 (44)	— (48)
	Female	78 (56)	— (52)
**Race, n (%)**			
	White	57 (41)	— (73)
	Hispanic	48 (34)	— (7)
	Black	32 (23)	— (21)
	Asian	3 (2)	— (2)
Baseline hemoglobin A_1c_ (%), mean (SD)		8.3 (2.5)	—
Hemoglobin A_1c_ readings per patient (%), mean (SD)		2.7 (0.9)	—
BMI (kg/m^2^), mean (SD)		33.6 (8.6)	—
Obesity, n (%)		85 (61)	— (32)
Hypertension, n (%)		104 (74)	— (37)
Smoking tobacco use, n (%)		49 (35)	— (35)

^a^—: not available.

**Table 2:** Hemoglobin A_1c_ goal^a^ achievement.

Time point	Controlled diabetes, n (%)	Uncontrolled diabetes, n (%)
Baseline	53 (100)	87 (0)
6 months	32 (85)	55 (20)
1 year	31 (77)	53 (25)
Most recent	53 (75)	87 (21)

^a^Proportion of patients who achieved a hemoglobin A_1c_ value of <7.0 at the aforementioned intervals relative to baseline. The sample size of each patient group is reported, followed by (in parentheses) the percentage of patients per sample achieving adequate glycemic control. For example, there were 55 patients with controlled diabetes who had hemoglobin A_1c_ data at 6 months from baseline. Of these 55 patients, 85% had a hemoglobin A_1c_ value of <7.0 at this time.

Glycemic control data were further stratified by race and Hispanic origin (Table 3). The clinic’s Hispanic population initially had the highest mean hemoglobin A_1c_ values among all demographic groups (8.6%) and had the greatest reduction in mean ΔA_1c_ (–0.6) across groups. White patients experienced the poorest response to treatment with a mean ΔA_1c_ of +0.2. The clinic’s Black population showed the highest rate of glycemic control (48% at baseline and 61% most recently).

Follow-up was measured as the percentage of patients in each group with a recorded hemoglobin A_1c_ value at 6 months and at 1 year. Patients with controlled diabetes had almost the same follow-up hemoglobin A_1c_ testing rates (63% at 6 months and 61% at 1 year) as patients with uncontrolled diabetes (61% at 6 months and 58% at 1 year). Follow-up rates did not differ significantly on the basis of gender, race, or Hispanic origin.

SLFMC data were compared to those of studies that similarly measured hemoglobin A_1c_ levels within target populations. The National Health and Nutrition Examination Survey (NHANES) generates samples with the intent of accurately representing the American adult population. Also included are 3 studies that reported posttreatment measures from student-run free clinics affiliated with 3 different medical schools. Rates of glycemic control at the SLFMC were superior to those observed at other free clinics and within 10% of 2 recently reported nationwide averages.

Finally, the rate at which the study group achieved controlled diabetes (hemoglobin A_1c_<7.0) was calculated at baseline and from the most recent available data (Table 4). Initially, 38% of the SLFMC patients had controlled diabetes; this proportion rose to 42% by the end of the study. The outcomes observed at the SLFMC were compared to similar hemoglobin A_1c_ data sets gathered from a literature review. Rates of control at the SLFMC were within 10% of two recent NHANES samples reported by Ali et al and Carls et al [31,32]. A more recent NHANES sample reported by Fang [33] showed a 56% rate of glycemic control. Beyond the nationwide data sets, the SLFMC outcomes were also compared to those published by three different student-run free clinics in various parts of the country. All these free clinic studies used similar methods and reported stratified posttreatment glycemic control data [12,13]. The glycemic control rate of the SLFMC exceeded all posttreatment hemoglobin A_1c_ control rates in the free clinic literature, but the SLFMC population displayed a lower mean hemoglobin A_1c_ at baseline than did the included clinics [12,13]. The stratified data reported from other free clinics represents posttreatment (rather than baseline) hemoglobin A_1c_ levels.

**Table 3:** Hemoglobin A_1c_ data stratified by race and Hispanic origin among patients at St. Luke’s Free Medical Clinic.

Metric	Non-Hispanic White (n=57)	Black (n=33)	Hispanic (n=47)
Hemoglobin A_1c_ value at baseline, mean	8.0	7.9	8.6
ΔA_1c_^a^, mean	+0.2	–0.3	–0.6
ΔA_1c_ in patients with controlled diabetes, mean (n)	+1.0 (22)	–0.3 (16)	+0.6 (14)
ΔA_1c_ in patients with uncontrolled diabetes, mean (n)	–0.4 (35)	–0.9 (17)	–1.1 (33)
Proportion of patients with a hemoglobin A_1c_ value of <7.0 at baseline, %	39	48	30
Proportion of patients with a hemoglobin A_1c_ value of <7.0 measured most recently, %	37	61	34

^a^ΔA_1c_: change in hemoglobin A_1c_ values; “+” indicates an increase and “–” indicates a decrease.

**Table 4:** Cross-comparison of hemoglobin A_1c_ data.

Hemoglobin A_1c_ value, %	SLFMC^a^ baseline measurement (N=140)	SLFMC most recent measurement (N=140)	NHANES^b^ 2007-2010 data (N=1444)	NHANES 2011-2014 data (N=1326)	NHANES 2011-2016 data (N=5800)	UCSD^c^ Free Clinic data (N=181)	Vanderbilt University Free Clinic data (N=45)	Icahn School of Medicine Free Clinic data (N=44)
<7.0, %	38	42	52	51	56	30	38	21
7.0-8.0, %	20	20	27	21	—^d^	29	24	18
>8.0, %	42	38	21	28	—	41	38	61
Hemoglobin A_1c_, mean	8.3	8.3	7.2	—	7.2	9.2	9.6	10.1
ΔA_1c_^e^, mean	–0.3	–0.3	—	—	—	–1.0	–1.7	–1.3
Most recent hemoglobin A_1c_ value	8.0	8.0	—	—	—	8.2	7.9	8.8

^a^SLFMC: St. Luke’s Free Medical Clinic.

^b^NHANES: National Health and Nutrition Examination Survey.

^c^UCSD: University of California San Diego.

^d^—: not applicable.

^e^ΔA_1c_: change in hemoglobin A_1c_ values.

## Discussion

### Principal Findings

In analyzing the follow-up behaviors of the SLFMC’s patients, we noted no significant difference in follow-up rates between patients with controlled and those with uncontrolled diabetes. However, there were some differences between the follow-up rates of different ethnic groups at the SLFMC. Prior research shows that Black and Hispanic patients tend to have higher baseline hemoglobin A_1c_ levels [34-37] and poorer self-management of diabetes, are less likely to achieve good glycemic control with treatment [38], and are more likely to experience diabetes-related complications resulting in worse diabetes outcomes and higher rates of mortality than non-Hispanic White patients [39]. Hispanic patients may specifically face obstacles associated with cultural differences between patients and providers, placing them at an increased risk for underuse of services, poor-quality care, and worse outcomes compared with non-Hispanic Whites [40]. The literature suggests that language barriers have a negative influence on health behaviors and outcomes among Latino patients [41]. To our knowledge, no unifying theory explains these known discrepancies in hemoglobin A_1c_ values among ethnic groups.

Black and Hispanic patients at the SLFMC defied the data cited above, with higher use of resources and better health outcomes than non-Hispanic White patients at the clinic (Table 3). Hispanic patients had a mean ΔA_1c_ of –0.6 while enrolled at the SLFMC. Black patients had a remarkable 61% glycemic control rate by the end of the study, which was the highest of any other race. SLFMC provides free care, including many free prescription medications, and we believe that ease of access to care is a primary driver of the trend-reversing outcomes achieved by the SLFMC’s Black and Hispanic patients with diabetes. Generally, Black and Hispanic populations are subject to disproportionately low health care access [42,43]. Furthermore, patient trust in providers is a direct predictor of subjective treatment effect [44,45]. The authors offer the following original thoughts on care for minorities within the free clinic setting: we suggest that patients are more likely to trust practitioners who provide their services free of charge. It is likely that patients feel more comfortable and less intimidated in the setting of charitable care compared to payment-based models. We also suggest that the ratio of immigrants in the Hispanic population is higher than that in other ethnicities [46]. We think that immigrants are more likely to use free health services out of appreciation for some of the things that American culture takes for granted. The aforementioned factors probably influenced the stronger improvements in hemoglobin A_1c_ values observed in Black and Hispanic patients than in Non-Hispanic White patients (Table 3).

Free clinic populations universally show hemoglobin A_1c_ averages at least a full point above nationwide averages for adults with diabetes (Table 4). The NHANES, which was chosen as the national comparison data set, generates annual samples with the intent of representing the American population as a whole. Most of the patients included in the NHANES sample have health insurance coverage; hence, NHANES suitably represents the glycemic control of the insured population. The SLFMC data are encouraging and suggest that free clinics may be able to approach the quality of care provided by traditional models of outpatient practices.

The glycemic control data gained in this study add to the growing body of knowledge characterizing the health of patients who utilize free clinic services. At baseline, The SLFMC population demonstrated the highest rate of controlled diabetes (38%) and the lowest mean hemoglobin A_1c_ value (8.3) observed in free clinic outcome literature, as well as the smallest mean improvement in hemoglobin A_1c_ values (–0.3). These findings are likely attributable to the relatively good health of the clinic’s population at the start the study. The COVID-19 pandemic occurred within the study timeline, and it probably influenced the modest mean improvement in hemoglobin A_1c_ value of –0.3. Altogether, this study presents the current best-case scenario of free clinic glycemic control at baseline.

Quantifying free clinic quality of care is a matter of importance to individual free clinics for the purpose of fundraising, but this aim is complicated by many factors. One such factor is time. The SLFMC has used paper charts for decades and has not completely transitioned to electronic medical records (EMRs). Many other free clinics are likely in the same situation because EMRs are costly to adopt. Sifting through paper charts takes more time than searching through a well-kept EMR. Another factor is available labor. Volunteer providers may not always be able to spare the time it takes to write up and publish outcomes. Despite obstacles of time and cost, the NAFC recently approved quality reporting standards, which it expects each member clinic to abide by. This presents a valuable professional growth opportunity for medical students and other postgraduate medical professionals. We recommend that free clinics take further initiative and publish outcomes when possible.

### Limitations

The retrospective cross-sectional design is limited in scope and cannot explain relationships between variables. Study data were extracted largely from paper charts and were subject to potential inaccuracies. The clinic is currently transitioning from a paper chart system to an EMR, and some charts were unavailable for evaluation. The SLFMC yielded a population of 140 patients with diabetes with a unique demographic profile that may not generalize well to other clinics. Furthermore, this study did not consider the specific diabetes treatment modalities provided to each patient (pharmaceuticals, nutrition counseling, home glucose monitoring, etc) which were excluded from the study because treatment varies widely across patients and providers. One final limitation is that not all patients had the same number of follow-up hemoglobin A_1c_ tests—such variations were possibly due to, for example, duration of enrollment (including loss to follow-up) and missed appointments.

### Conclusions

This report highlights a free clinic diabetes treatment program that provides significant benefits to patients with uncontrolled T2DM. The SLFMC was successful in treating minority patients, with uncontrolled Black and Hispanic patients showing a mean 1% improvement in hemoglobin A_1c_ values. Rates of glycemic control at the SLFMC were superior to those observed at other free clinics and within 10% below two recently reported nationwide averages. This study presents one of the largest sample sizes yet observed in the free clinic hemoglobin A_1c_ literature. Limitations include loss to follow-up and incomplete patient records. Meta-analysis of comparable outcomes data is the intuitive next step in the journey toward a better understanding of the quality of care provided at free clinics.

## Abbreviations

△A_1c_: net change in hemoglobin A_1c_
ADA: American Diabetic Association
EMR: electronic medical record
NAFC: National Association of Free Clinics
NHANES: National Health and Nutrition Examination Survey
SLFMC: St. Luke’s Free Medical Clinic
T2DM: type 2 diabetes mellitus
